# Integrating Embodied Social Presence Theory and Process Virtualization Theory to assess business process virtualizability: The mediating role of embodied co-presence

**DOI:** 10.1371/journal.pone.0305423

**Published:** 2024-06-14

**Authors:** Juan Yang, Hualong Fang

**Affiliations:** 1 Department of Global Business, Chonnam National University, Yeosu, South Korea; 2 College of Liberal Arts, Cheongju University, Cheongju, South Korea; Alexandru Ioan Cuza University of Iasi, Faculty of Philosophy and Social-Political Sciences, ROMANIA

## Abstract

In the digital era, the integration of technology within business processes is pivotal for organizational efficiency. This study investigates the impact of IT characteristics-specifically IT representation, IT reach, and monitoring capability-on the virtualizability of business processes, utilizing the frameworks of Embodied Social Presence Theory (ESPT) and Process Virtualization Theory (PVT). Our objective is to explore how these IT characteristics, through the mediation of embodied co-presence, enhance business process virtualizability in the context of collaborative tools. Addressing a gap in existing literature, we propose that beyond facilitating virtualization, IT characteristics deeply influence virtual processes by integrating human-centric experiences of co-presence. To examine this, we analyzed data from 311 Korean employees with remote work experience during the COVID-19 pandemic using Smart-PLS 4. Our findings indicate that IT representation and IT reach significantly contribute to business process virtualizability, mediated by embodied co-presence. Surprisingly, monitoring capability did not significantly affect either embodied co-presence or process virtualizability, challenging initial assumptions. This study bridges ESPT and PVT to offer new insights into the roles of IT characteristics in process virtualization, highlighting the importance of human-centric IT design. The results provide valuable guidance for businesses and developers of collaborative tools, underscoring the need to enhance virtual work environments through technology that fosters a sense of presence and collaboration.

## Introduction

In the landscape of modern business, digital transformation has become a cornerstone for enterprise innovation, with the integration of collaborative tools marking a key milestone in this journey [1–4]. A critical aspect of this transformation is the enhanced virtualizability of business processes, a concept that encapsulates the potential and suitability of transitioning business processes from physical to virtual environments [5, 6]. In this context, PVT has gained prominence as a comprehensive framework for understanding the dynamics of business process virtualizability [5, 7, 8].

However, with the continual evolution and iterative updates of Information Systems (IS) like collaborative software, new research perspectives have shifted focus. Beyond the basic functionalities of IS, the emphasis is now on the more experiential aspects that these systems provide, such as ‘embodied co-presence,’ which encompasses interactive and sensory experiences for users [9, 10]. This change signifies a move towards understanding how IS can enrich human interactions and perceptions in digital environments. While many studies have confirmed that IT characteristics directly enhance process virtualizability, the in-depth exploration of the indirect mechanisms, particularly the mediating roles that facilitate this enhancement, remains largely unexplored. This lacuna in research not only limits theoretical advancement but also poses practical challenges for businesses and developers of collaborative tools, hindering their ability to effectively manage and leverage these technologies.

To bridge this gap, our study proposes an integrative approach, combining the insights of ESPT with PVT. This fusion aims to investigate the mediating mechanisms by which IT characteristics, as conceptualized within PVT, can promote the virtualizability of business processes. By incorporating the human-centric aspects of ESPT, particularly the nuances of embodied co-presence, our research offers a unique perspective on the interplay between technological capabilities and the experiential elements of virtual collaboration.

The methodology of this research primarily focuses on empirical analysis, supported by a detailed review of ESPT and PVT to inform our hypothesis development and research model. Drawing upon existing literature, we have adapted operational definitions and measurement scales, focusing our data collection on a survey conducted among Korean employees who have navigated remote work during the COVID-19 pandemic. The study analyzes responses from 311 participants, employing Smart-PLS 4 software for statistical analysis to validate the structural model and to rigorously test the proposed hypotheses.

This research significantly contributes to the existing knowledge base, offering a refined understanding of how IT characteristics influence the business processes virtualizability. The theoretical fusion of ESPT and PVT in this study elucidates the mechanisms through which collaborative tools can be optimized to enhance process virtualizability. Importantly, it highlights the mediating role of embodied co-presence, thereby laying a foundation for more effective management and utilization of these technologies in an increasingly digitalized business world. This integration not only advances our theoretical understanding but also guides practical applications in optimizing virtual collaboration.

## Theoretical background

### Embodied Social Presence Theory

ESPT has become increasingly relevant in the context of the digital era, particularly within workplace settings and remote work environments. This theory centers around the concepts of presence and interaction within virtual spaces, addressing how individuals engage with and perceive their environment and others in digital contexts [11, 12].

Central to ESPT is the concept of embodiment within virtual environments, often realized through avatars or digital representations [13, 14]. This embodiment plays a crucial role in creating a sense of “being there” and, more importantly, “being together” with others in virtual settings [15–17]. Known as embodied co-presence, this aspect of ESPT extends beyond an individual’s sense of personal presence in a virtual space to include the feelings of connectedness and interaction with others [14, 18, 19]. It significantly impacts collaboration and team dynamics, especially in remote work scenarios where effectively replicating the nuances of face-to-face interactions can greatly enhance team cohesion and productivity [10, 20].

Technologies that enhance embodied co-presence can bridge physical distance, facilitating more effective and engaging virtual collaborations [21, 22]. This becomes especially crucial as businesses continue to adapt to a digital-first landscape, underscoring the need for advanced virtual communication tools that support complex, dynamic work environments. These technologies foster a stronger sense of togetherness and improve communicative clarity, enhancing the sense of co-presence and, consequently, the overall effectiveness of virtual collaborations [23].

The principles of ESPT advocate for creating virtual workspaces that support embodied co-presence. This involves designing digital environments that replicate aspects of physical interactions to improve communication and collaboration in remote settings [24]. By enhancing the sense of co-presence, organizations can foster more engaging and interactive virtual work experiences, leading to improved employee engagement and team cohesion.

In summary, ESPT provides valuable insights into virtual interactions in the workplace, particularly under remote work conditions. It addresses the absence of physical cues and direct face-to-face interactions, proposing that a robust sense of co-presence can significantly enhance communication and collaboration among remote teams [25]. The theory offers a framework for enhancing virtual work experiences, aiming to improve communication, collaboration, and overall job satisfaction in digital environments.

In the context of our study, embodied co-presence offers a novel lens through which to understand how IT characteristics contribute to the virtualizability of business processes. It highlights that enhancing business process virtualizability is not only a matter of technological capacity but also involves how these technologies shape the user experience in terms of co-presence. IT features that foster a strong sense of embodied co-presence can lead to more effective virtual collaborations and interactions, crucial for the successful virtualization of business processes.

### Process virtualization theory

PVT offers a comprehensive framework for understanding the transformation of business processes through digital means. Originally proposed by Eric Overby in 2008 [8], PVT has evolved into a critical tool in assessing how traditional, physical processes can be effectively replicated and executed in a virtual environment [5, 7, 8, 26]. Central to PVT is the concept of virtualizability, which explores the extent to which business processes can function effectively in a virtual setting, minimizing the reliance on physical resources or face-to-face interactions. This concept is instrumental in navigating the complexities of digital transformation, helping businesses to identify which processes are most amenable to virtualization and predicting the challenges that might arise during this transition.

Overby’s foundational work laid the groundwork for PVT, introducing key constructs like sensory, relationship, synchronization, and identification/control requirements. These constructs are crucial for determining the feasibility of virtualizing specific processes, with sensory requirements addressing the extent to which physical senses need to be simulated virtually, and relationship requirements focusing on the importance of personal interactions in the process execution. Synchronization requirements consider the need for real-time or sequential activities, while identification and control requirements involve the ability to verify identities and manage process controls effectively.

His research highlights the roles of IT characteristics, such as representational fidelity, reach, and monitoring capability, in facilitating process virtualization [8, 26]. Representational fidelity ensures that the virtual processes accurately mimic the physical ones, reach extends the accessibility of the process to a wider audience, and monitoring capability allows for the oversight and management of the process remotely. Together, these IT characteristics enhance the efficiency and effectiveness of virtualized processes, supporting organizations in their efforts to adapt to an increasingly digital business landscape.

Building upon this foundation, recent studies have expanded the application and understanding of PVT across various domains. For example:

Feng et al. (2023) examined the barriers to business process virtualization in remote work, particularly highlighting how sensory and synchronization requirements reduce effectiveness. The study identifies critical challenges in mimicking real-time interactions and managing sensory inputs virtually, essential for efficiency in remote settings. It also discusses the psychological strain on teleworkers and issues like communication overload, suggesting the need for strategies to overcome these barriers for better virtualization post-COVID-19 [5].

Alarabiat et al. (2023) explored how virtualization requirements influence student satisfaction in online learning, using PVT. Their study highlights the significant impacts of sensory and relationship needs on student engagement and satisfaction. These findings illustrate the importance of addressing these specific virtualization requirements to enhance educational outcomes in online settings [27].

In the public sector, Ackom et al. (2022) investigated the application of PVT in e-government services in developing countries. Their study highlights the uneven virtualization potential across different governmental processes, pointing out that not all processes are equally amenable to virtualization. This research underscores the importance of a selective approach in applying virtualization strategies to government operations, suggesting that alignment with user acceptance and process characteristics is crucial for effective implementation [7].

In the domain of human resources, the application of PVT has been explored to understand the dynamics of e-HR processes. Specifically, Yeh and Hsiao (2017) focused on how relationship requirements and monitoring capabilities influence the acceptance of e-HR processes. Their study revealed that certain virtualization requirements significantly affect user acceptance and operational efficiency in HR settings. By analyzing the relationship and monitoring aspects of virtual HR processes, their research provides valuable insights into how organizations can enhance their HR operations through virtual platforms. This extension of PVT into human resources highlights the potential for tailored virtualization strategies that can improve both the efficiency and effectiveness of HR services [28].

These contributions collectively enrich the PVT framework, demonstrating its versatility across various sectors including remote work, online education, e-governance, and retail banking. They emphasize the evolving challenges of virtualization and the importance of sector-specific strategies for effective implementation. In summary, PVT continues to be an essential tool in managing digital transformation, providing businesses with valuable insights into which processes are best suited for virtualization and aiding in the navigation of challenges during the transition from physical to digital workflows.

### Integration of ESPT and PVT

This research explores the intricate relationship between IT characteristics and business process virtualizability by integrating insights from PVT and ESPT. The primary aim is to reveal how IT features-specifically IT representation, IT reach, and monitoring capability-impact business process virtualizability, with a special focus on the mediating role of embodied co-presence from ESPT.

In PVT, IT Representation is about how closely IT can mimic physical processes in a virtual environment. Survey feedback highlighted the importance of receiving accurate and comprehensive information through collaboration tools, which is essential for replicating the nuances of physical processes virtually. IT Reach reflects the extent of IT’s influence across an organization. The effectiveness of collaboration tools in facilitating teamwork, regardless of physical location, underpins IT’s expansive role in bridging functional silos within businesses. Monitoring Capability involves IT’s ability to oversee business activities. Responses underscored the significance of features within collaboration tools that allow for the tracking of tasks and the efficient management of workflows, crucial for maintaining oversight in a virtual setting [5, 7, 8, 26–28].

While recognized as vital for virtualization, the mechanisms through which these IT aspects affect virtualization are often underexplored.

ESPT brings a crucial perspective, focusing on the perceived presence in a virtual environment and its impact on virtual collaboration. Embodied co-presence is hypothesized to be the bridge connecting IT characteristics with the effectiveness of virtualized business processes [9, 25]. For instance, better IT representation can create more realistic virtual environments, enhancing co-presence, which then leads to more efficient process virtualization.

To further elucidate, embodied co-presence not only enriches the user experience by fostering a sense of community and collaboration in virtual settings but also serves as a critical factor in the seamless integration of digital processes [29, 30]. This sense of presence and togetherness, despite geographical separations, is instrumental in overcoming the inherent challenges of virtual teamwork, such as communication barriers and feelings of isolation [31]. Moreover, the degree to which individuals feel co-present in digital environments can significantly influence their engagement levels, decision-making processes, and overall productivity within virtualized business operations [32].

We propose that IT features boost embodied co-presence, facilitating effective virtual collaborations and improving business process virtualizability. This novel perspective combines the technical aspects of PVT with the human-centric focus of ESPT, offering a holistic view of how IT attributes not only support but also enrich the quality of virtualized processes through enhanced social dynamics.

In summary, this integration of ESPT and PVT provides new insights into the depth of IT’s impact on business process virtualization. It shifts the focus to include social and perceptual dimensions, offering a deeper understanding of how IT infrastructure can elevate the virtual work experience, leading to improved communication, collaboration, and job satisfaction.

## Research model and hypothesis development

### Research model

Our research model, conceptualized at the intersection of PVT and ESPT, examines the impact of IT characteristics (IT representation, IT reach, monitoring capability) on business process virtualizability. The model positions IT characteristics as independent variables, embodied co-presence as a mediating variable, and business process virtualizability as the dependent variable.

Integrating the technological insights from PVT with the human interaction focus of ESPT, we hypothesize that IT characteristics within an organization influence business process virtualizability both directly and indirectly. This indirect influence is mediated through the enhancement of embodied co-presence, suggesting that robust IT infrastructure not only supports but also enriches the quality of interactions in virtual environments, crucial for the effective virtualization of business processes.

**Fig 1:**
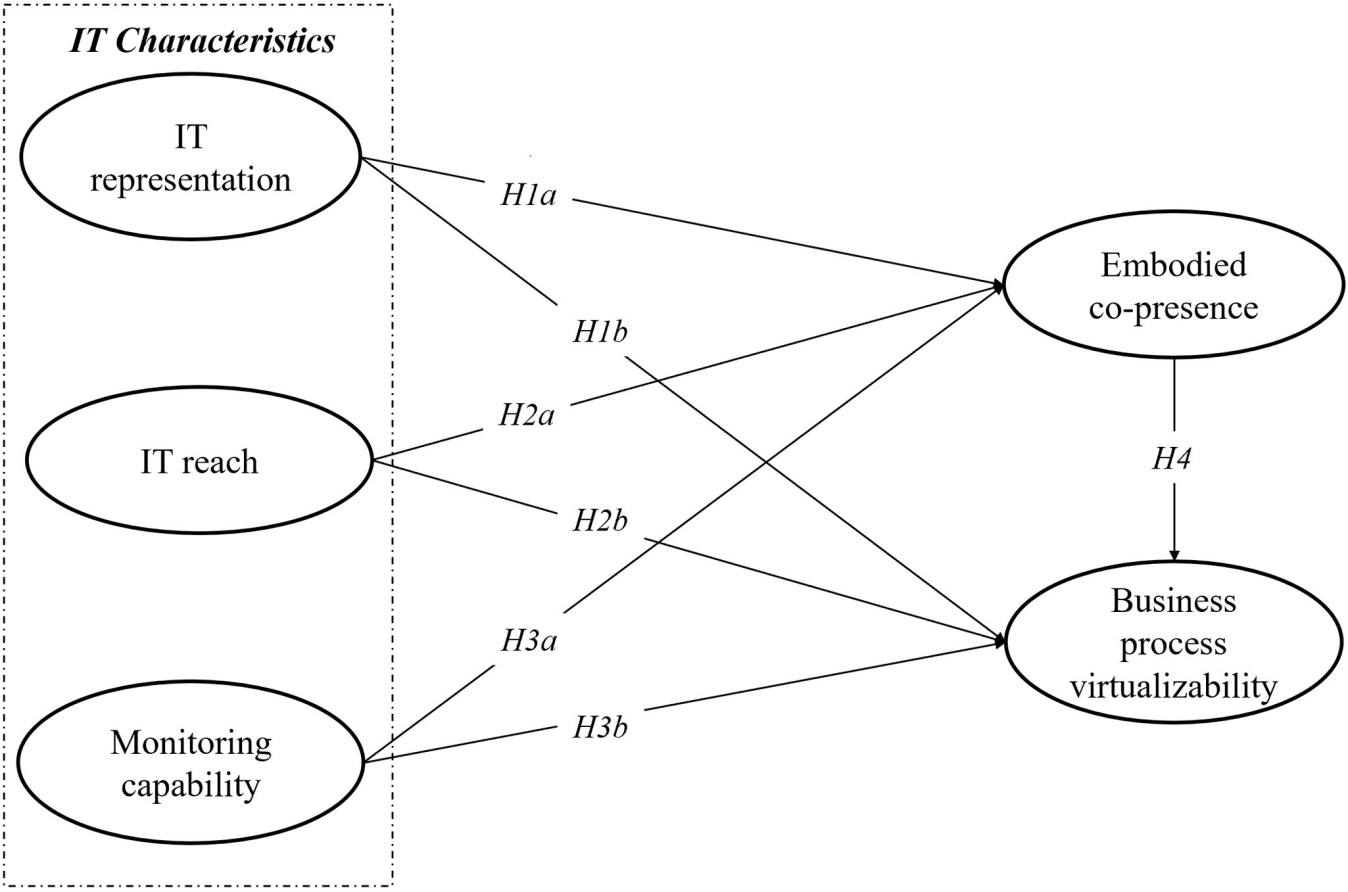
Research model.

This dual pathway, combining direct and indirect impacts, forms the core of our model, as illustrated in Fig 1. It aims to elucidate how the synergy between technology infrastructure and human interaction dynamics can transform traditional business processes into efficient virtual operations.

### Hypothesis development

IT Representation refers to how information technology is integrated into and represented within an organization’s processes and infrastructure. It encompasses the deployment of IT resources, tools, and systems that support business operations, such as communication, collaboration, and data management. Effective IT representation implies a comprehensive and functional integration of technology into the organizational framework [8].

Embodied co-presence is a fundamental element of ESPT. It encapsulates the sense of presence and togetherness individuals experience in virtual environments [33–36]. This perception of being alongside others, notwithstanding physical separation, is paramount in the realm of virtual collaborations and remote work settings [37, 38]. The essence of embodied co-presence is intrinsically tied to the quality and effectiveness of the virtual environment, which, in turn, is substantially influenced by the organization’s IT infrastructure.

By intertwining IT representation with embodied co-presence, we assert that an organization’s thorough and effective IT representation significantly enhances virtual interactions and environments. This enhancement cultivates a deeper sense of embodied co-presence among users. Advanced IT tools and platforms are instrumental in this context, as they forge more immersive and interactive virtual experiences—critical components for nurturing a feeling of co-presence. This aspect gains heightened relevance in today’s digital collaboration landscape, where virtual presence, facilitated by technology, compensates for the lack of physical interactions.

Reflecting on empirical research in virtual communication and collaboration, we observe that the technological medium—specifically, the caliber and capacity of IT representation—plays an indispensable role in molding users’ experiences of co-presence in virtual domains. This correlation is echoed in studies that link sophisticated IT infrastructure to improved experiences in virtual interactions [39, 40].

Consequently, we formulate the following hypothesis:

***H1a***: ***IT representation has a positive impact on embodied co-presence*.**

Moving forward, we delve into the concept of business process virtualizability, which is the extent to which an organization’s processes can be executed effectively in a virtual or digital environment. Influenced by the nature of the process, available technology, and the organization’s readiness for virtualization, this concept determines the feasibility of transitioning physical processes to digital platforms [8, 27].

Linking these two concepts, we can argue that enhanced IT representation leads to better integration of digital tools and processes, crucial for virtualizing business processes. In an era marked by a surge in remote work and digital transformation, IT representation becomes increasingly significant. Advanced IT infrastructure and tools not only facilitate remote access and collaboration but also enable business processes to adapt to and thrive in digital environments [41–44]. Furthermore, a robust IT framework ensures accessible and manageable data and workflows, which are key to the virtualizability of business processes [45, 46].

Empirical and theoretical support for this linkage is substantial. Research in virtual collaboration and remote work indicates that advanced IT tools significantly enhance the quality of virtual interactions and process execution, fostering the virtualizability of business processes [47]. Additionally, theoretical frameworks, such as PVT, emphasize the crucial role of technological mediums (i.e., IT representation) in enabling process virtualization [8, 26, 48].

Considering the discussed theoretical and empirical evidence, we are led to the formulation of a critical hypothesis in the domain of digital transformation and organizational technology management. This hypothesis, derived from the interplay between IT representation and business process virtualizability, is succinctly captured as follows:

***H1b***: ***IT representation has a positive impact on business process virtualizability*.**

Within the framework of PVT, IT reach is defined as the extent to which information technology systems and networks within an organization extend across and integrate various functional areas, facilitating communication and resource sharing [8, 26]. This concept encompasses not just the physical infrastructure of IT but also the scope of its applicability across different organizational segments. IT reach determines the efficiency with which information is disseminated and operations are coordinated, especially in a distributed or virtual setting [49].

The hypothesis asserts that an expansive IT reach positively impacts the sense of embodied co-presence. This is predicated on the idea that when IT infrastructure provides extensive and seamless connectivity, individuals in a virtual environment experience a stronger sense of presence and connection with others [50–53]. This enhanced sense of co-presence is vital in virtual environments for effective collaboration, communication, and overall engagement.

In today’s digital-centric work and social environments, where virtual interactions are increasingly common, the role of IT reach becomes even more pronounced. Organizations with a wide-reaching and integrated IT infrastructure are likely to facilitate better virtual interactions, which is crucial for maintaining the social dynamics and collaborative spirit in virtual settings [50–53].

Thus, based on the theoretical foundations provided by PVT and ESPT, along with the practical considerations of modern IT infrastructure’s role in virtual environments, we propose:

***H2a***: ***IT reach has a positive impact on embodied co-presence*.**

The virtualization of business processes entails moving traditional, often physically bound, business operations to a digital or virtual platform. This transition requires seamless connectivity and a robust IT infrastructure that can bridge physical distances and functional divides [8]. An extensive IT reach implies a comprehensive network that connects various components of an organization, enabling efficient data exchange and communication [54]. In a virtualized environment, this interconnectedness is vital for process coherence, real-time data access, and operational agility.

The hypothesis posits that a greater IT reach within an organization positively impacts the virtualizability of its business processes. This is because an expansive IT reach enhances the organization’s ability to conduct operations remotely or digitally, without the constraints of physical location or traditional communication barriers. A broad IT reach supports the integration of disparate systems and processes, facilitating a smoother transition to and functioning of virtualized processes [55, 56]. It enables the leveraging of digital tools and platforms essential for modern, virtual operations.

In the current digital-driven business landscape, the significance of IT reach in process virtualization has been further underscored. The shift towards remote work models, global operations, and digital service delivery models necessitates an IT infrastructure that can support wide-reaching, seamless, and efficient virtual operations. Organizations with a high IT reach are better equipped to adapt to these evolving business paradigms and maintain operational efficiency in a virtual context.

Based on these theoretical and practical considerations, we propose the following hypothesis.

***H2b***: ***IT reach has a positive impact on business process virtualizability*.**

In the realm of PVT, monitoring capability encapsulates the IT infrastructure’s effectiveness in overseeing virtual processes from employees’ perspectives, ensuring quality and adherence to standards [8]. This capability is crucial for maintaining oversight and control over virtualized processes, ensuring that they are efficient, effective, and aligned with organizational goals. Monitoring capability encompasses not just the collection of data but also its analysis and the provision of actionable insights.

The effectiveness of monitoring capability is hypothesized to extend beyond process management and into enhancing the quality of virtual interactions. This capability, which encompasses the oversight, analysis, and reporting of activities in virtual spaces, is crucial for maintaining the integrity and effectiveness of these environments. When monitoring systems are adept at ensuring smooth operational flow, they inadvertently contribute to a heightened sense of presence and interaction, key components of embodied co-presence.

In virtual environments where collaboration and interaction are pivotal, robust monitoring ensures minimal disruptions and optimal functioning of communication and collaboration tools [57]. This reliability and quality assurance provided by effective monitoring contribute to a stronger sense of being present and engaged in these virtual settings [58]. It is this aspect of monitoring capability that is proposed to positively influence the embodied co-presence. Individuals are more likely to feel a profound sense of connection and engagement in a well-monitored and seamlessly functioning virtual environment.

Therefore, we posit that there is a direct positive impact of monitoring capability on the sense of embodied co-presence in virtual environments:

***H3a***: ***Monitoring Capability has a positive impact on embodied co-presence*.**

The inherent value of monitoring capability, especially from the vantage point of employees engaged in virtual environments, is fundamental to the successful virtualization of business processes. This capability extends beyond traditional oversight; it’s about enabling a transparent, responsive, and adaptable digital workspace [59]. Effective monitoring systems are not merely tools for control but pivotal elements that bridge the gap between physical operations and their virtual counterparts. They ensure that even in the absence of a centralized, physical workspace, quality, compliance, and operational efficiency are not compromised.

In the evolving landscape of digital operations, where virtual workspaces are becoming the norm, the demand for robust monitoring capabilities has intensified. Such capabilities allow organizations to maintain a pulse on their virtual processes, facilitating swift adaptations to changes, streamlining workflows, and upholding stringent quality standards. This adaptability and oversight are critical for organizations aiming to thrive in dynamic and often complex virtual environments [60].

Considering the strategic importance of monitoring capability, especially from the perspective of employees who rely on it to enhance their virtual work experience, we propose a nuanced hypothesis:

***H3b***: ***Monitoring Capability has a positive impact on business process virtualizability*.**

Proceeding further, we explore how embodied co-presence positively influences the business process virtualizability. This exploration integrates the concept of embodied co-presence from ESPT with PVT’s principles of process virtualization.

Embodied co-presence a fundamental element of ESPT, denotes the perceived sense of connection and collaboration with others in a virtual environment [14, 18]. This concept becomes particularly relevant in scenarios where traditional physical interactions are supplanted by virtual engagements, such as in remote work and digital teamwork [14, 18, 61].

Our hypothesis asserts that an enriched experience of embodied co-presence can significantly enhance the business process virtualizability. This enhancement stems from the premise that effective and engaging virtual collaboration is crucial for the seamless transition and execution of business operations in a virtual domain. A strong sense of co-presence not only facilitates more effective communication and collaboration but also serves as a catalyst for virtualizing business processes more efficiently [62].

In today’s rapidly evolving work environment, characterized by increasing remote operations and virtual team structures, the role of embodied co-presence gains heightened importance. For organizations looking to excel in virtual settings, cultivating a robust sense of co-presence among team members is key. It not only promotes smoother and more productive interactions but also plays a pivotal role in enhancing the adaptability and efficiency of business processes in the virtual landscape.

Therefore, marrying the insights from ESPT with the operational focus of PVT, we articulate the following hypothesis:

***H4***: ***Embodied co-presence has a positive impact on business process virtualizability*.**

## Research methodology

This study integrates ESPT and PVT to explore how IT characteristics-specifically IT representation, IT reach, and monitoring capability-influence business process virtualizability through the enhancement of embodied co-presence.

IT representation in PVT refers to how information technology is integrated into and depicted within an organization’s processes. It encompasses the deployment and utilization of IT resources, tools, and systems in facilitating various business operations [8].

IT reach pertains to the extent and effectiveness with which information technology can extend the capabilities of an organization. This includes the ability of IT to connect various components of the organization, facilitate communication and data exchange, and enable access to resources across different locations and platforms [8].

Monitoring capability involves the ability to oversee, manage, and control processes within a virtual environment. It includes the tools and methodologies used to track performance, manage workflows, and maintain quality control in a virtual setting [8].

The constructs in this study were measured using a 5-point Likert scale, ranging from “Not at all” to “Very much so.” Table 1 details the measurement items for each construct.

**Table 1:** Questionnaire.

Variables		Item	Source
Independent Variable	IT representation	The collaboration tool consistently delivers the information I need.	Overby (2008) Ofoeda et al. (2018) [63]
		I find the information provided by the collaboration tool to be comprehensive and sufficient for my needs.	
		The collaboration tool effectively keeps me informed about my boss and colleagues.	
		The quality of information I receive through the collaboration tool is suitable for my job requirements.	
	IT reach	The collaboration tool supports inclusive participation, enabling adequate involvement from everyone in the team.	
		The collaboration tool effectively facilitates tasks that necessitate teamwork.	
		The collaboration tool offers features that promote collaboration among team (or department) members.	
		The collaboration tool is versatile, allowing me to manage work tasks from any location and at any time.	
	Monitoring capability	The collaboration tool I use effectively tracks the progress of tasks and projects.	
		I find the monitoring features of our collaboration software to be efficient in managing workflows.	
		I can easily monitor the status of ongoing work using our virtual collaboration platform.	
Mediator Variable	Embodied co-presence	While using collaborative tools, I experience a sense of others’ presence around me.	Poeschl and Doering (2015) Zhang et al. (2022)
		When engaged in digital workspaces, I feel that my presence is noticeable to others.	
		In virtual collaborative environments, I feel a sense of companionship, not isolation.	
Dependent variable	Business process virtualizability	I like working in an online virtual environment.	Overby (2008) Feng et al. (2023) Ofoeda et al. (2018) [63]
		I think it would be better to handle tasks in an online virtual environment that I would usually handle in an offline face-to-face environment.	
		I prefer working remotely in a virtual environment based on collaboration tools.	
		I think it’s good to work remotely using a collaboration tool.	

The study conducted a survey among 330 professionals who have experience with remote work or work-from-home, facilitated by collaboration tools. The survey, which ran from March 2023 to present, resulted in 311 valid responses after excluding insincere replies. The sampling error for the results, calculated at a 95% confidence level, is estimated to be approximately 5.7%.

The methodology for this research was carefully designed to uphold the highest standards of ethical integrity and comply with local and institutional requirements. We ensured that all participants were fully informed about the aims of the survey and their rights as participants. Written informed consent was obtained from each participant, guaranteeing their voluntary participation in the study.

To further protect the participants, we made a clear commitment to anonymity and confidentiality right from the outset of our survey. In accordance with Article 13 of the Statistical Law (Protection of Secrets), we included a statement at the beginning of the survey, emphasizing that: “This survey is conducted anonymously. All data collected will be kept confidential and securely protected under Article 13 of the Statistical Law (Protection of Secrets). The information gathered will be exclusively used for statistical analysis and will not be disclosed for any purposes beyond this study.”

This approach ensured that participants felt safe to provide honest responses, aware that their identity would remain anonymous and their information confidential.

Table 2 presents the descriptive statistics of the study’s sample population, providing a detailed breakdown of participant demographics and their use of various collaboration tools. The table encompasses data from a total of 311 respondents, representing a comprehensive cross-section of individuals with diverse backgrounds in terms of gender, age, education level, and the types of collaboration tools used.

**Table 2:** Descriptive statistics.

Variable		No. of people	Proportion
All		311	100.00%
Gender	Male	145	46.60%
	Female	166	53.40%
Age	20–30	51	16.40%
	30–40	142	45.66%
	40–50	98	31.51%
	>50	20	6.43%
Education Level	High school diploma or lower	21	6.75%
	Associate degree	39	12.54%
	Bachelor’s degree	220	70.74%
	Graduate degree or higher	31	9.973%
Collaboration tools used (multiple selections possible)	Zoom	219	51.41%
	Microsoft 365	58	13.62%
	Naver Works	55	12.91%
	Proprietary Collaboration Platform	43	10.09%
	Slack	26	6.10%
	Kakao work	25	5.87%

## Data analyses and results

This study employs Structural Equation Modeling (SEM) to investigate the relationships posited in our hypotheses. SEM is chosen for its robustness in examining complex models and its ability to simultaneously test multiple relationships [64]. The analysis will be conducted using Smart PLS (Partial Least Squares), a software tool known for its effectiveness in dealing with smaller sample sizes and complex models.

The constructs in our study-IT representation, IT reach, monitoring capability, embodied co-presence, and business process virtualizability-will be measured using validated scales from existing literature. These scales will be adapted slightly to fit the context of our study while maintaining their original validity. Respondents will rate items on a Likert scale, providing quantitative data for analysis.

SEM, as implemented through Smart PLS, will be used to test the relationships between the constructs in our model. Smart PLS is particularly suited for this study due to its ability to handle complex models and its robustness in path modeling with latent variables [64]. The PLS approach to SEM is also preferred for its minimal demands on measurement scales and distributional assumptions, making it suitable for exploratory research.

The analysis will proceed in two stages. The first stage involves assessing the measurement model to ensure reliability and validity of the constructs. This includes checks for Cronbach’s alpha, composite reliability, convergent validity, and discriminant validity. The second stage involves assessing the structural model to test the proposed hypotheses. This will include evaluating path coefficients and significance levels [65, 66].

### Measurement model result

This research employs Smart-PLS 4 as the analytical tool to validate the study’s model. The Partial Least Squares (PLS) analysis process necessitates the validation of internal consistency, convergent validity, and discriminant validity of the measurement items and constructs [66, 67].

To verify internal consistency, the analysis focused on rho_A, rho_C for Composite Reliability (CR), the Average Variance Extracted (AVE), and Cronbach’s alpha. As indicated in Table 3, the results demonstrate that Cronbach’s alpha values are above the 0.7 threshold, suggesting acceptable levels of reliability. Similarly, both rho_A and rho_C for CR are above 0.7, and AVE values exceed 0.5 [68, 69]. These findings indicate that each measurement variable exhibits high internal consistency, which is crucial for ensuring the reliability of the constructs within our model.

**Table 3:** Construct reliability and validity.

Variable	Cronbach’s alpha	rho_a	rho_c	AVE	loadings	t-values
Business process virtualizability	0.862	0.875	0.915	0.783	0.906	88.959
					0.843	57.326
					0.904	44.062
Embodied co-presence	0.895	0.895	0.935	0.826	0.909	78.575
					0.914	89.268
					0.904	73.791
IT reach	0.906	0.906	0.934	0.780	0.910	20.564
					0.867	27.746
					0.860	50.969
IT representation	0.877	0.890	0.924	0.802	0.933	84.348
					0.886	27.252
					0.867	70.567
Monitoring capability	0.749	0.757	0.856	0.664	0.809	21.253
					0.845	27.411
					0.790	18.263

In the process of affirming convergent validity within our study, a comprehensive approach was undertaken. Initially, confirmatory factor analysis was employed as a means to scrutinize the individual factor loadings of each construct. Subsequent to this, a rigorous bootstrapping analysis was performed, focusing on evaluating the statistical significance of these loadings, as suggested in prior research [70, 71]. This analysis yielded compelling results: each construct demonstrated predominant factor loadings when compared to others, with loadings consistently exceeding the 0.7 benchmark [72]. Furthermore, the analysis of t-values associated with these factor loadings revealed that all values were comfortably above the critical threshold of 1.96. This is indicative of their statistical significance at a 5% level [73]. Such outcomes robustly support the convergent validity of our model, showcasing that each construct effectively captures the essence of the variables it is intended to measure.

Finally, the discriminant validity of our model was assessed by examining whether the square root of the Average Variance Extracted (AVE) for each construct is greater than the correlation coefficients between the constructs, as recommended in the literature [70]. As per the results presented in Table 4, the lowest value of the square root of AVE (0.814) is higher than the highest correlation coefficient (0.532) among the constructs. This outcome confirms that the constructs in our proposed model are sufficiently distinct from each other, thereby demonstrating good discriminant validity.

**Table 4:** Discriminant validity.

Variable	1	2	3	4	5
1.Business process virtualizability	0.884				
2.Embodied co-presence	0.466	0.908			
3.IT reach	0.400	0.076	0.883		
4.IT representation	0.332	0.374	0.070	0.895	
5.Monitoring capability	0.449	0.261	0.532	0.061	0.814

Considering the analyses conducted thus far, our study’s measurement model satisfies the criteria for internal consistency, convergent validity, and discriminant validity. Based on these established standards, we proceeded to the subsequent phase of Structural Equation Modeling (SEM) analysis, aimed at verifying the relationships between the hypotheses and the research variables.

### Structural model result

This study initiated a comprehensive analysis to evaluate the proposed structural model and its implications. Our analytical approach was multifaceted, encompassing a diagnosis of collinearity and the estimation of path coefficients. These components were integral in thoroughly assessing the model’s performance and validity. Each element of this analysis contributed significantly to our understanding of the model’s robustness, ensuring that the findings are both reliable and reflective of the underlying theoretical constructs.

An essential step in our structural model analysis was to check for multicollinearity, which we approached through the Variance Inflation Factor (VIF). We calculated the VIF values for all predictors in the model to ensure that the variables did not exhibit problematic collinearity. According to the results presented in Table 5, all VIF values fell below the threshold of 3.3, as recommended in the literature [74]. This outcome is critical as it confirms the absence of multicollinearity issues among the predictor variables, thereby underlining the stability and reliability of our structural model. These findings allow us to confidently proceed with the analysis and interpretation of the variable relationships, free from concerns about multicollinearity affecting the study’s conclusions.

**Table 5:** Collinearity statistics (VIF).

Variable	Business process virtualizability	Embodied co-presence
Business process virtualizability		
Embodied co-presence	1.221	
IT reach	1.055	1.021
IT representation	1.135	1.024
Monitoring capability	1.053	1.002

In our analysis using the PLS structural model, the path coefficients play a pivotal role. These coefficients are indicators quantifying the strength of the relationships between the independent and dependent variables [67, 75]. In this study, the PLS methodology was utilized to calculate the path coefficients among the independent variables (IT reach, IT representation, Monitoring capability), the mediator variable (Embodied co-presence), and the dependent variable (Business process virtualizability). These path coefficient values are instrumental in understanding the influence and weight of each relationship in the model.

To establish the statistical significance of these path coefficients, we employed the bootstrapping technique within PLS, executing it 5,000 times. This rigorous process allowed us to accurately determine the t-values associated with each path coefficient, providing a robust measure of their statistical significance [76].

Furthermore, we conducted an analysis of specific indirect effects to validate the mediating effect of the mediator variable, embodied co-presence. This part of the analysis was crucial for understanding how embodied co-presence potentially intermediates the relationship between our independent and dependent variables, thereby offering deeper insights into the underlying dynamics of our research model.

The analysis of the structural model, as presented in Table 6, reveals insightful findings about the relationships hypothesized in our study. The outcomes are as follows:

Hypothesis H1a (IT representation → Embodied co-presence): This hypothesis is supported with a β-value of 0.336 and a t-value of 5.891, indicating that IT representation significantly influences embodied co-presence.
Hypothesis H1b (IT representation → Business process virtualizability): Also supported, showing a positive relationship with a β-value of 0.180 and a t-value of 2.933. This suggests that IT representation effectively enhances business process virtualizability.
Hypothesis H2a (IT reach → Embodied co-presence): Supported with a β-value of 0.287 and a t-value of 5.430, confirming that IT reach positively impacts embodied co-presence.
Hypothesis H2b (IT reach → Business process virtualizability): This hypothesis is supported, as indicated by a β-value of 0.219 and a t-value of 4.168, demonstrating that IT reach contributes positively to business process virtualizability.
Hypothesis H3a (Monitoring capability → Embodied co-presence): Not supported, evidenced by a β-value of 0.102 and a t-value of 0.952. This indicates that monitoring capability does not have a statistically significant impact on embodied co-presence.
Hypothesis H3b (Monitoring capability → Business process virtualizability): Similarly, this hypothesis is not supported, with a β-value of 0.023 and a t-value of 0.261, suggesting that monitoring capability does not significantly influence business process virtualizability.
Hypothesis H4 (Embodied co-presence → Business process virtualizability): Supported with a β-value of 0.319 and a t-value of 6.859. This strongly indicates that embodied co-presence has a positive impact on business process virtualizability.

**Table 6:** Path coefficient.

Hypothesis	Relationship	β-value	t-value	Decision
H1a	IT representation → Embodied co-presence	0.336	5.891	Supported
H1b	IT representation → Business process virtualizability	0.180	2.933	Supported
H2a	IT reach → Embodied co-presence	0.287	5.430	Supported
H2b	IT reach → Business process virtualizability	0.219	4.168	Supported
H3a	Monitoring capability → Embodied co-presence	0.102	0.952	Not Supported
H3b	Monitoring capability → Business process virtualizability	0.023	0.261	Not Supported
H4	Embodied co-presence → Business process virtualizability	0.319	6.859	Supported

These results provide a clear understanding of the various influences IT characteristics and embodied co-presence have on the virtualizability of business processes. They highlight the significant roles played by IT representation and IT reach, while also underscoring the importance of embodied co-presence in enhancing the virtualizability of business processes.

### Mediation effect

In our structural model, we examined the mediation effect of embodied co-presence in the relationships between IT characteristics (IT representation and IT reach) and business process virtualizability. The results, as detailed in Table 7, provide insights into the strength and significance of these mediated relationships [77].

**Table 7:** Assessment of mediation effect.

Path	β	STDEV	T statistics	P values
IT representation -> Embodied co-presence -> Business process virtualizability	0.120	0.026	4.658	0.000
IT reach -> Embodied co-presence -> Business process virtualizability	0.116	0.026	4.422	0.000
Monitoring capability -> Embodied co-presence -> Business process virtualizability	0.027	0.022	1.205	0.228

The path from IT representation to business process virtualizability, mediated by embodied co-presence, shows a path coefficient (β) of 0.120. With a standard deviation (STDEV) of 0.026 and T statistics of 4.658, this path is statistically significant (P value = 0.000). This indicates a substantial and positive mediation effect of embodied co-presence in the relationship between IT representation and business process virtualizability.

The path involving IT reach to business process virtualizability, mediated by embodied co-presence, exhibits a path coefficient of 0.116. The statistical significance of this relationship is confirmed by T statistics of 4.422 and a P value of 0.000, with a standard deviation of 0.026. This indicates a significant positive mediation effect of embodied co-presence on the impact of IT reach on business process virtualizability.

For the path from monitoring capability to business process virtualizability, mediated by embodied co-presence, the results present a different scenario. The path coefficient here is 0.027, with T statistics at 1.205 and a P value of 0.228. This higher P value and lower T statistics indicate that this particular mediation effect is not statistically significant.

In summary, the mediation analysis demonstrates that embodied co-presence significantly mediates the impact of IT representation and IT reach on business process virtualizability. However, for monitoring capability, the mediation effect through embodied co-presence is not statistically significant. These insights are crucial in understanding the differentiated roles played by various IT characteristics in enhancing business process virtualizability through the intermediary of embodied co-presence.

## Discussion

This study illuminates the complex interplay between IT characteristics and business process virtualizability, particularly through the lens of digital collaboration tools. A key finding is the significant impact of IT representation and IT reach on enhancing process virtualizability. These elements—effective information integration (IT representation) and the facilitation of connectivity and resource accessibility (IT reach)—are crucial for augmenting virtualization capabilities in business operations. Our results not only align with but also expand the current literature on digital transformation by emphasizing the need for a comprehensive and integrated IT infrastructure to support efficient virtual operations.

Moreover, our research introduces a novel element by exploring the mediating role of embodied co-presence in linking IT characteristics to business process virtualizability. The study reveals that both IT representation and IT reach are instrumental in strengthening embodied co-presence, which in turn positively affects process virtualizability. This mediation underscores the importance of human experience within virtual environments, building on prior research to show how a sense of cohesiveness and collaboration in digital realms can enhance the operational effectiveness of virtual business processes.

Contrary to our initial hypotheses and prevailing narratives in existing research, which often highlight the critical role of monitoring capability in virtual efficiency, our findings suggest its influence on both embodied co-presence and business process virtualizability is minimal. This unexpected result prompts a reevaluation of the traditional understanding of monitoring capability’s role in virtual settings. Although monitoring tools are vital for ensuring operational oversight and maintaining quality, their effectiveness in fostering a sense of co-presence and enhancing virtualizability seems less significant compared to other IT characteristics. This revelation not only challenges conventional wisdom but also opens new avenues for research into the nuanced dynamics between monitoring capabilities and virtual collaboration effectiveness.

By integrating these insights into the existing body of knowledge on digital collaboration and process virtualization, our study not only enriches academic discourse but also sets forth new pathways for future inquiry. It encourages a deeper investigation into how various IT characteristics distinctly contribute to and interact within the virtualization of business processes, highlighting an evolving digital work environment where technology and human experience merge to redefine efficiency and connectivity.

### Practical contributions

The findings from this study illuminate key areas for practical application within the realms of business operations and the development of collaboration tools. For organizations aiming to refine their digital presence, the emphasis on IT representation and reach emerges as a strategic imperative. This insight guides businesses in molding their digital transformation efforts to not only accommodate but to enhance the virtualizability of their processes. It suggests a shift towards creating digital workspaces where interactions are not just transactions, but experiences enriched by a sense of connection and presence.

Developers of collaboration tools are encouraged to look beyond the functional to the experiential, focusing on how their technologies can foster a sense of co-presence. This entails crafting digital environments that are not merely platforms for task completion but spaces where engagement and interaction mirror the richness of face-to-face collaboration. Such a perspective pivots the development of these tools towards enhancing the human experience, making virtual collaboration not just effective but also meaningful.

In doing so, both organizations and tool developers can contribute to a digital workspace ecosystem that leverages technology to enhance human connectivity and collaboration, paving the way for more dynamic and effective virtual operations.

### Theoretical contributions

This study makes a significant theoretical contribution by seamlessly integrating ESPT and PVT. It highlights the pivotal role of embodied co-presence as a mediator, demonstrating how IT characteristics like IT representation and IT reach, traditionally emphasized in PVT for their technical utility, also enhance the social dynamics of virtual work environments. This interdisciplinary approach enriches our understanding of virtual collaboration, merging the technical aspects of IT infrastructure with the human-centric perspective of virtual presence.

Our research expands the scope of IT characteristics in PVT, underscoring their impact on the social interaction within virtual environments. The findings challenge existing notions about monitoring capability, suggesting its influence on embodied co-presence and business process virtualizability may be more nuanced than previously thought. This reevaluation invites future research to explore the contextual dependencies of IT features in virtual settings, offering new directions for investigating the complex interplay between technology and social presence in digital workspaces.

Overall, this study contributes to a deeper understanding of how technology characteristics and human experiences interact to enhance virtual work processes. It underscores the need for a balanced approach that combines technical efficiency with a focus on human-centric design in digital collaboration tools.

### Limitations and future research

While our study provides valuable insights into the integration of Artificial Intelligence (AI) into Information Systems (IS) for enhancing virtual workspaces, it also encounters certain limitations. These include the scope of AI technologies assessed, the diversity of virtual work environments considered, and the potential variability in user experiences across different sectors. Recognizing these limitations is crucial for framing the context in which our findings should be interpreted.

Looking ahead, the evolving role of AI in redefining virtual collaboration and process virtualization presents a fertile ground for further exploration. Future research should delve into how AI technologies can enhance user experiences, streamline virtual processes, and introduce innovative methods of collaboration. Such investigations are essential for advancing our comprehension of digital transformation and for informing the development of more dynamic and engaging virtual work environments. This prospective research avenue promises to extend our study’s findings by addressing its limitations and exploring new dimensions of AI’s impact on virtual workspaces.

## Conclusion

In summarizing the findings of this study, we unveil nuanced insights into the synergy between IT characteristics and the virtualizability of business processes, a relationship significantly deepened through the integration of ESPT with PVT. The impact of IT representation and IT reach on enhancing the adaptability of business processes to virtual environments has been underscored, highlighting the indispensable role of embodied co-presence as a critical mediator. This revelation elevates the discourse beyond the mere technical competencies of IT, emphasizing the value of human-centric considerations within digital workspaces.

Unexpectedly, our investigation into the monitoring capability presented a twist in our narrative, revealing a less significant influence on virtualization than originally posited. This outcome nudges us toward a reflective reevaluation of its perceived utility in digital collaboration landscapes, suggesting a complex, context-dependent impact that merits further exploration.

Delving into the practical realm, this research extends its tendrils across various sectors, underscoring the applicability of our findings in shaping digital transformation strategies. The insights gleaned beckon organizations to strategically prioritize IT features that not only promise technical robustness but also foster a sense of connection and collective endeavor in virtual domains. From enhancing customer engagement in the service industry through immersive virtual experiences to empowering the educational sector with broader access and interactional richness, and even redefining patient care in healthcare through more empathetic telemedicine practices, the implications are vast and varied.

This journey into the intersection of technology attributes and human experiences within virtual collaboration unfolds a roadmap for navigating digital transformations more effectively. It beckons future inquiries into how these insights could be tailored and applied across different sectors, enriching our collective understanding of digital collaboration’s evolving dynamics. Therein lies the invitation for continued exploration, to dissect the sector-specific nuances of these findings and further our grasp on optimizing virtual operations in an increasingly digitized era.

In essence, this study not only contributes a layered perspective to the academic discourse surrounding digital transformation but also charts a course for organizations aiming to refine their virtual operational strategies. It heralds a new chapter in understanding the multifaceted nature of technology and human interaction within the business process virtualizability, offering a beacon for future research and practical application alike.
